# Improvement of Naturally Derived Food Colorant Performance with Efficient Pyranoanthocyanin Formation from *Sambucus nigra* Anthocyanins Using Caffeic Acid and Heat

**DOI:** 10.3390/molecules25245998

**Published:** 2020-12-18

**Authors:** Nicole Straathof, M. Monica Giusti

**Affiliations:** Department of Food Science & Technology, The Ohio State University, Columbus, OH 43210, USA; straathof.2@osu.edu

**Keywords:** elderberry, natural food color, pigment, color stability, color intensity, CIELab, uHPLC, spectrophotometry

## Abstract

Consumers and regulations encourage the use of naturally derived food colorants. Anthocyanins (ACN), plant pigments, are unstable in foods. In aged red wines, ACN with a free hydroxyl group at C-5 condenses to form pyranoanthocyanins (PACN), which are more stable but form inefficiently. This study attempted to produce PACN efficiently using high cofactor concentration and heat. Elderberry anthocyanins were semi-purified and caffeic acid (CA) was dissolved in 15% ethanol and diluted with a buffer to achieve ACN:CA molar ratios of 1:50, 1:100, 1:150, and 1:200, then incubated at 65 °C for 5 days. The effect of temperature was tested using ACN samples incubated with or without CA at 25 °C, 50 °C, and 75 °C for 7 days. Compositional changes were monitored using uHPLC-PDA-MS/MS. Higher CA levels seemed to protect pigment integrity, with ACN:CA 1:150 ratio showing the highest tinctorial strength after 48 h. PACN content growth was fastest between 24 and 48 h for all ACN:CA ratios and after 120 h, all ACN had degraded or converted to PACN. PACN formed faster at higher temperatures, reaching ~90% PACN in 24 h and ~100% PACN in 48 h at 75 °C. These results suggest that PACN can form efficiently from elderberry ACN and CA if heated to produce more stable pigments.

## 1. Introduction

People experience colors daily that not only affect their feelings, but also their decisions. Food color is an important characteristic that has been used for over a century to help consumers identify and differentiate product safety, sensory, and quality characteristics [1]. However, many consumers do not trust artificial ingredients and look for natural alternatives. In one study, scientists determined that artificial colorants increase hyperactivity in children [2]. This study raised concerns and lead numerous countries to ban specific artificial food colorants.

Naturally derived alternatives have become an important area of research, with anthocyanins (ACN) receiving a fair share of attention. These compounds are plant pigments found in many plants that act as powerful antioxidants and may provide health benefits. Unfortunately, they are unstable and lose their vibrancy over time. Therefore, scientists asked: What is a food that has color for a very long time? The answer was found in wine. Scientists studying wine found pyranoanthocyanins (PACN), which are anthocyanin-derived pigments.

Pyranoanthocyanins were first discovered in red wine in 1996 as a crucial component of wine color [3,4]. That same year, the structure was determined through NMR and showed the pyran ring attached between the C-5 hydroxyl group and C-4 [5]. Because of the additional pyran ring, pyranoanthocyanins have a **λ**_max_ that is hypsochromically shifted and expresses a more orange color compared to authentic anthocyanin pigments [4]. This additional ring makes PACN a much more stable pigment that is better suited for food processing environments [6]. Unfortunately, they are formed very inefficiently over time. As shown in wine, color development from PACN can take months to years [7]. In a food processing setting, storing the pigment for that amount of time is a cost not many are willing to spend. Increasing the formation rate of PACN is a relevant area of research. PACN require anthocyanins, a reaction partner, and storage time for formation [4]. PACN formation in food is dependent on the concentration of both the anthocyanins and the reaction partner. In wine, PACN form and cause color changes through aging, which can take months through to decades. Because of the increased stability of this pigment, studies look for ways to increase the rate of formation. Pinotin A, a hydroxyphenyl-pyranoanthocyanin, was shown to have the fastest formation in Pinotage wine. In this time period of 3 to 4 years, concentration of anthocyanins had significantly decreased, leading to an increase in the caffeic acid to anthocyanin ratio [8]. One study looked at using microwave-assisted extraction to increase PACN formation at differing grape ripeness levels [9]. Another study was able to reduce PACN formation to 42 days by increasing storage temperature to 25 °C [10]. However, with increased temperature, anthocyanin degradation also increases. The benefit of gaining a more stable pigment needs to be weighed with the loss of the initial pigment. Nonetheless, the efficient formation of PACN would be an asset to the food industry as they work towards eliminating artificial food colorants.

It has been shown that increasing storage temperature from refrigeration to room temperature can increase PACN formation; however, temperatures beyond room temperature have not been evaluated [10,11]. These studies also found that caffeic acid is an effective cofactor in producing PACN. However, excessive caffeic acid used without obtaining significant increases in PACN formation would be a costly waste of product for producers. Additionally, too much caffeic acid may cause astringent flavor development [12]. Determining the amount of caffeic acid that is beneficial to the development of PACN without sacrificing other characteristics could save producers money as they switch to naturally derived colorants.

This study consisted of two main objectives: to determine if using high incubation temperature would increase PACN formation significantly and to determine if using a higher concentration of cofactor and temperature would increase PACN formation enough for use as a naturally derived colorant. With the addition of significantly more heat, it is hypothesized that PACN formation and anthocyanin degradation will both increase. Furthermore, we expected that the increase in caffeic acid concentrations with a high incubation temperature would increase PACN formation, but that it may not be significant.

## 2. Results and Discussion

**Figure 1:**
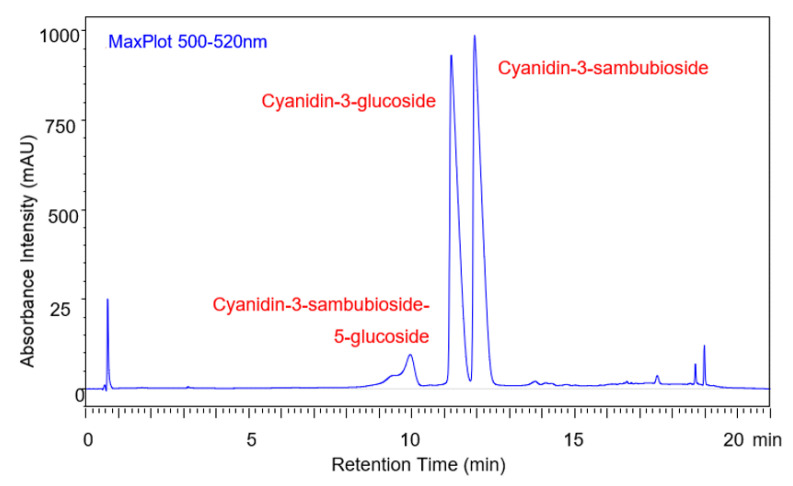
Elderberry anthocyanins before incubation with caffeic acid. uHPLC-PDA-MS/MS settings were as follows: 0.25 mL/min flow, 50 °C oven temperature, 50 μL injection volume, and the following acetonitrile gradient: 0% at 0 min, 26% at 19 min, 30% at 20 to 21 min, 0% at 21 min.

There were three main anthocyanins eluted from the sample including cyanidin-3-glucoside, cyanidin-3-sambubioside, and cyanidin-3-sambubioside-5-glucoside. The identification of cyanidin-3-glucoside and cyanidin-3-sambubioside confirmed that the elderberry sample was an ideal anthocyanin source for PACN formation. The free hydroxyl group at the C5 position of these main anthocyanins allowed for a cofactor to condense and form PACN. These results agreed with literature by Lee and Finn, which found that the first two small anthocyanin peaks are cyanidin-3-sambubioside-5-glucoside and cyanidin-3,5-diglucoside, respectively [13]. They determined that the next two larger anthocyanin peaks were cyanidin-3-sambubioside and cyanidin-3-glucoside, respectively. MS/MS data were used for pigment identification. A large portion of the sample was made up of peaks two and three (cyanidin-3-glucoside and cyanidin-3-sambubioside) (Figure 1).

With the main anthocyanins in this sample having a free hydroxyl group at the C5 position, this sample was an ideal candidate for PACN formation. When the sample was analyzed using the uHPLC-MS/MS-PDA methods described in the methods section, our results agreed with those described previously by Lee and Finn. The two peaks that eluted around 18 min were the PACN peaks, but when the sample was not incubated, the amount was very minimal. It was, however, present in the sample containing caffeic acid at low concentrations because of the time that passed between sample preparation and analysis.

**Figure 2:**
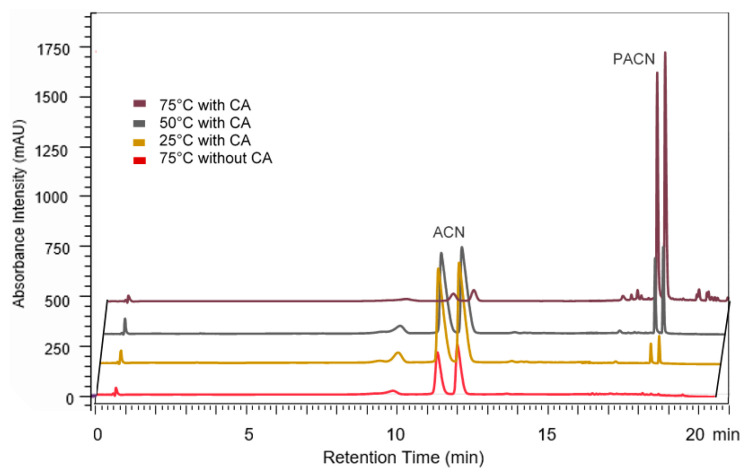
Retention time of two main anthocyanins (ACN) and pyranoanthocyanins (PACN) obtained from sample incubated for 24 h at 75 °C with and without caffeic acid (CA), 50 °C with caffeic acid, and 25 °C with caffeic acid. Chromatograms viewed between 500 and 520 nm. uHPLC-PDA-MS/MS settings were as described in Figure 1.

After 24 h incubation at higher temperatures, the anthocyanin peak areas decreased faster, while the PACN peak areas increased faster over time than at lower temperatures (Figure 2). The reverse was true at lower temperatures, with greater anthocyanin peak area and smaller PACN peak areas after 24 h of incubation.

**Figure 3:**
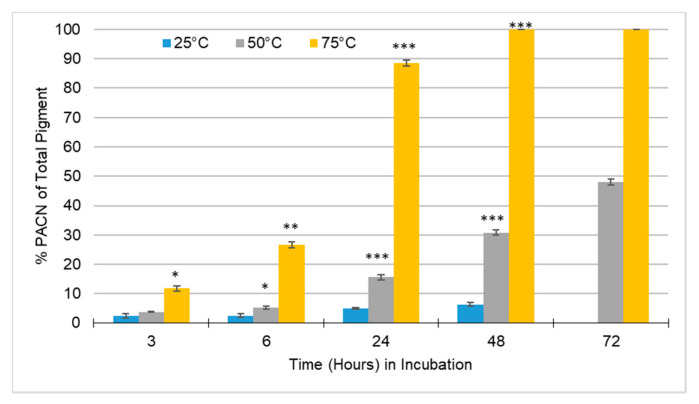
Average percentage of pyranoanthocyanin (PACN) of total pigment of samples with caffeic acid incubated over time at 25 °C, 50 °C, and 75 °C. Calculated by Equation (4). Sample incubated at 25 °C for 72 h was not measured because of minimal PACN present and sample loss. Error bars represent the standard deviations. Significant difference from 25 °C incubation for the same incubation time with * *p* < 0.05; ** *p* < 0.01; *** *p* < 0.001.

By comparing PACN formation and anthocyanin degradation, it can be seen that at lower temperatures the anthocyanin degraded less but formed less PACN (Figure 2). On the contrary, at higher incubation temperatures, PACN formation was greater and anthocyanin degradation was greater. Therefore, past a specific time, incubation was counterproductive because it will not increase PACN significantly while continuing to degrade anthocyanin pigments consistently. Figure 3 showed a significant increase in PACN formation at 75 °C compared to 25 °C and 50 °C.

Within 24 h, there was 86% PACN and within 48 h the sample was 100% PACN. Incubating beyond 48 h decreased total pigment. Because of anthocyanin sensitivity to heat, degradation of pigment is expected at high temperatures [14,15]. According to Figure 3, it would be nearly pointless to incubate beyond 48 h under these conditions. Even though PACN is more stable, at 75 °C incubation there may still be minimal pigment loss. At 48 h of incubation under such a high heat, the greatest concentration of PACN has already been reached and continued incubation leads to unnecessary loss of pigment and wasted energy.

**Figure 4:**
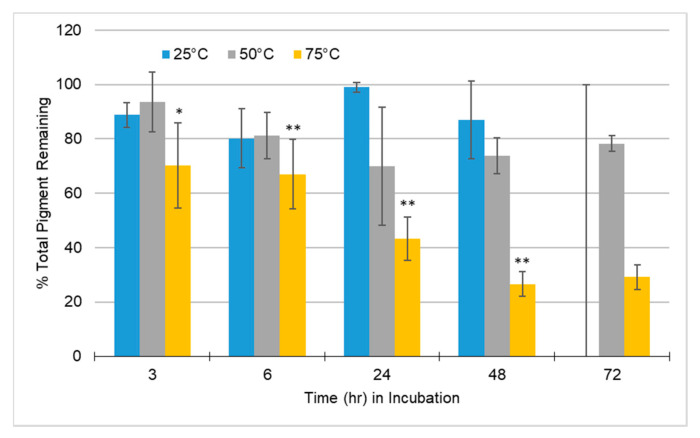
Average percentage of total pigment remaining in samples incubated with caffeic acid at 25 °C, 50 °C, and 75 °C over time; 25 °C at 72 h was not measured because of minimal pyranoanthocyanin present and sample loss. Calculated with Equation (5). Error bars represent the standard deviations. Significant difference from 25 °C incubation for the same incubation time with * *p* < 0.05; ** *p* < 0.01.

In the samples analyzed, at higher temperatures, degradation of total pigment occurred more rapidly than at lower temperatures (Figure 4).

Looking at the results shown in Figure 4, it could be argued that the production of PACN would be more efficient at 24 h versus 48 h because of the significant difference between 6 and 24 h versus the minimal difference between the former (Figure 3). The processing cost of storing and incubating the sample combined with the loss in pigment remaining may not be worth the minimal increased PACN content. Although pigment was lost through the incubation of sample and formation of PACN, the amount of PACN present in the sample was comparatively substantial. A similar study incubating samples at 25 °C required 42 days to obtain the PACN levels obtained within one day in this study at 75 °C [10]. Being able to produce PACN within one day versus several weeks or months increases the practicability of producing PACN in the food industry as a naturally derived alternative to artificial colorants.

**Figure 5:**
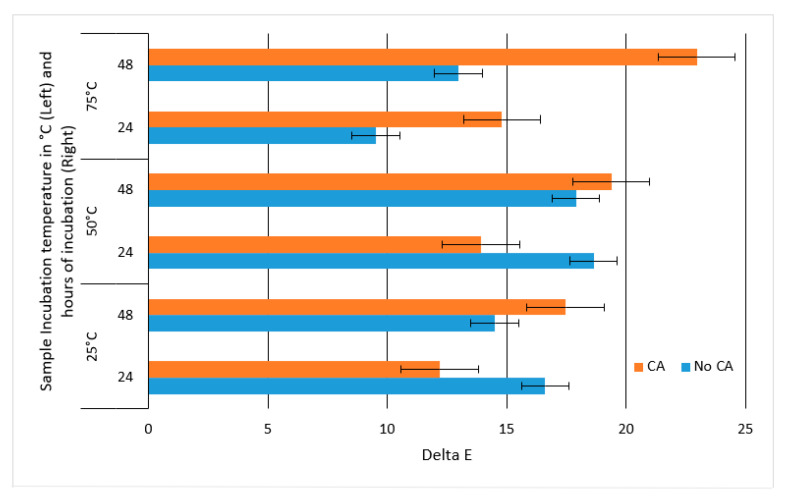
Average ΔE*_ab_ of samples incubated at 25, 50, or 75 °C between samples incubated for zero hours and either 24 or 48 h with or without caffeic acid (CA). Error bars represent the standard deviations.

Because of the loss of anthocyanins and the formation of PACN, a color change occurred in the sample. According to Figure 5, at 25 °C and 50 °C, the ΔE*_ab_ was greater at 48 h than 24 h with caffeic acid. At 75 °C, the delta E increased with caffeic acid between both 24 and 48 h when compared to no incubation.

Figure 5 showed that with the formation of PACN the ΔE*_ab_ increased, indicating a change in the pigment. Each ΔE*_ab_ was greater than five. This pigment could therefore not replace anthocyanins, but it may be used in tandem with them.

**Figure 6:**
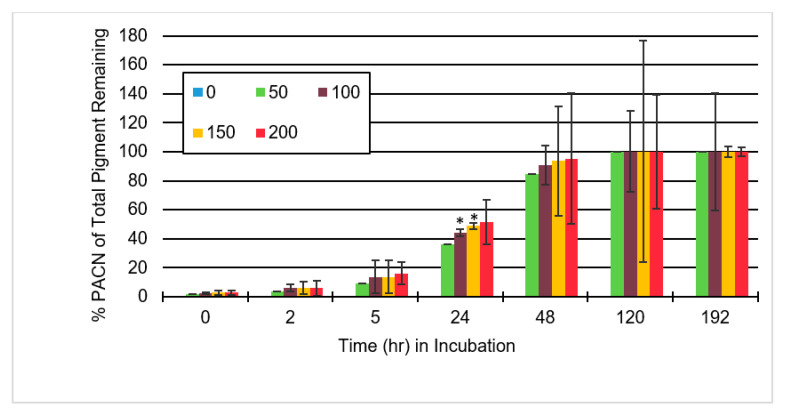
Average total pyranoanthocyanin (PACN) as part of total pigment after time in incubation at 65 °C with samples containing anthocyanin to caffeic acid molar ratios of 1:0, 1:50, 1:100, 1:150, and 1:200. Error bars represent the standard deviations. Significant difference from anthocyanin to caffeic acid molar ratio of 1:50 for the same incubation time with * *p* < 0.05.

The PACN content increased consistently with an increase in caffeic acid concentration, but it did not increase significantly (Figure 6). Therefore, under the conditions tested it may not be worth adding more than an ACN:CA molar ratio of 1:50.

Because Figure 6 shows a consistent but not a significant increase in PACN present, the addition of more caffeic acid may have more of a negative effect than the benefits it provides. Too much caffeic acid may cause astringent flavor development and may become a costly investment for producers. Within 48 h, samples containing caffeic acid had all achieved PACN concentrations of at least 80%, while at 120 h all samples contained 100% PACN. With at least 80% PACN in the sample with ACN:CA molar ratios of 1:50, the sample may already contain enough pigment and have enough tinctorial strength for what is required.

**Figure 7:**
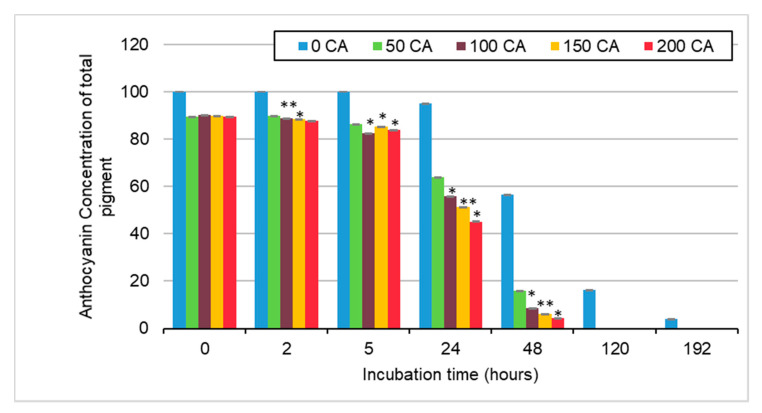
Average percentage of anthocyanin of total pigment in sample containing anthocyanin to caffeic acid molar ratios of 1:0, 1:50, 1:100, 1:150, and 1:200 after hours of incubation at 65 °C. Error bars represent the standard deviations. Significant difference from ACN:CA molar ratio of 1:50 for the same incubation time with * *p* < 0.05, ** *p* < 0.01.

In Figure 7 we can see that through incubation, anthocyanin concentration decreased over time. We can also see that the anthocyanin concentration decreases more rapidly at higher concentrations of caffeic acid.

This would suggest that much of the loss of anthocyanins is attributed to the formation of PACN. As is also shown in Figure 6, we can see that between 24 and 48 h there was a significant (*p*-value < 0.05) shift from anthocyanins to PACN in samples containing caffeic acid. Some time after 48 h, anthocyanins were either completely degraded or converted to PACN, as a sample with no caffeic acid was the only sample with anthocyanins remaining at 120 h.

**Figure 8:**
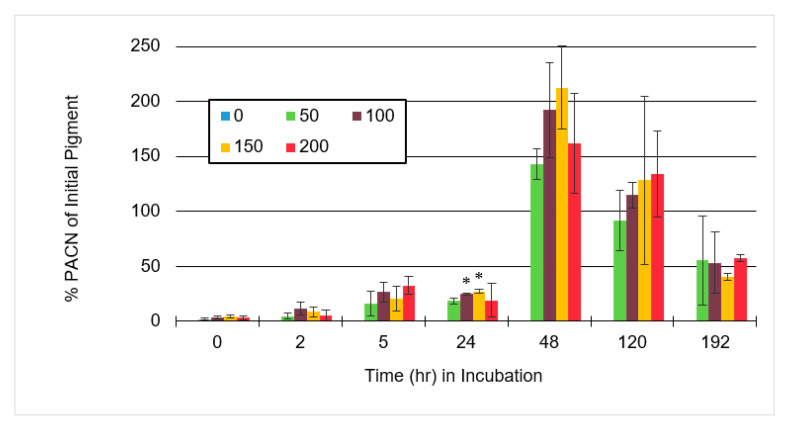
Average pyranoanthocyanin (PACN) concentration of initial pigment concentration in a sample containing anthocyanin to caffeic acid molar ratios of 1:0, 1:50, 1:100, 1:150, and 1:200 after hours of incubation at 65 °C. The sample without caffeic acid was not observed because there was no PACN present. Error bars represent the standard deviations. Significant difference from anthocyanin to caffeic acid molar ratio of 1:50 for the same incubation time with * *p* < 0.05.

Figure 8 showed a sample with ACN:CA molar ratios of 1:150 and 48 h of incubation had the greatest PACN conversion at 212% absorbance compared to the initial pigment.

At this time of incubation, PACN absorbance was greater than 125% of the initial sample for each caffeic acid containing the sample. This showed that with the use of caffeic acid, the amount of color expression in samples incubated for 48 h could be greater with PACN formation than the pigment in the initial sample. These results are solely based on absorbance, specifically the area under the curve for anthocyanins and relative concentration. Results could not be compared to a standard curve because of the lack of PACN standard samples. With greater color expression and stability, the pigment may be used to create and provide more reliable colors for use in the food industry. Sometime after 48 h, degradation overtook formation and total pigment decreased, meaning that under these conditions incubating past 48 h was not worth the loss of pigment, unless a pure sample of PACN is the goal.

## 3. Materials and Methods

### 3.1. Materials

Standardized organic elderberry (*Sambucus nigra*) was obtained from Artemis International, Inc. (Fort Wayne, IN, USA). The following chemicals were sourced from Fisher Scientific (FairLawn, NJ, USA): ethyl acetate, methanol, HPLC-MS grade acetonitrile, HPLC-MS grade water, and hydrochloric acid. Acetic acid and caffeic acid were sourced from Millipore Sigma (Darmstadt, Germany). Formic acid was purchased from EMD Millipore Corporation (Danvers, MA, USA).

### 3.2. Methods

#### 3.2.1. Preparation of Elderberry Anthocyanin Extract

Standardized elderberry powder was weighed (1.2 g) and solubilized in 1800 mL of distilled water. Sample was semi-purified using a C18 resin (Waters Corporation, Milford, MA, USA) through solid phase extraction. The column was activated with methanol and rinsed with three volumes of acidified water. The sample was added to the column until it was packed halfway, then two volumes of acidified water and three volumes of ethyl acetate were added. Anthocyanins were then eluted using three volumes of MeOH and collected. Methanol was evaporated out using a Buchi Rotovapor (Buchi Corporation, New Castle, DE, USA) with a 35 °C water bath. The remaining semi-purified solution was resolubilized in 0.01% acidified water. The sample was stored at −18 °C until use. This process was repeated twice to produce a total of three replicates.

#### 3.2.2. Determining Concentration of Monomeric Anthocyanins in Extract

Concentration of monomeric anthocyanins in extract was determined according to the pH Differential method provided by Giusti and Wrolstad [16]. Sample was diluted separately with both potassium chloride buffer at pH 1.0 and sodium acetate buffer at pH 4.5 at a dilution factor where the **λ**_max_ was below 1 for the sample diluted with potassium chloride buffer. The dilution factor was calculated by dividing the final volume of the sample with the final diluted volume of the sample and absorbance was measured on the SpectraMax M2 spectrophotometer (Molecular Devices, Sunnyvale, CA, USA). Once both the pH 1.0 and pH 4.5 sample dilutions were equilibrated for 15 min, the spectrophotometer was used to measure absorbance at the **λ**_max_ and 700 nm against distilled water. The following calculation was used to determine the absorbance (A), which was used in the latter equation to determine the monomeric anthocyanin pigment concentration.

A = (A_λ__vis-max_ − A_700 nm_) _pH 1.0_ − (A_λ__vis-max_ − A_700 nm_) _pH 4.5_ (1)

Monomeric anthocyanin pigment (mg/liter) = (A × MW × DF × 1000) ÷ (ε × 1) (1)(2)

A = Absorbance, MW = molecular weight of predominant anthocyanin, DF = Dilution factor, ε = Molar absorptivity of predominant anthocyanin.

#### 3.2.3. Preparation of Caffeic Acid and Anthocyanin Sample

To measure the effect of temperature, caffeic acid was added to 100% ethanol in Eppendorf tubes (Eppendorf, Hamburg, Germany), which were centrifuged for 5 min. The aliquots were removed, and these steps were repeated twice. This was used as a saturated caffeic acid in ethanol solution and 11 mL of EtOH with or without caffeic acid was added to 62.5 mL Phosphate Citrate buffer and 22.5 mL ACN in Phosphate Citrate buffer.

To measure the effect of caffeic acid concentration, the weight of caffeic acid required in each sample was calculated using the molar mass of cyanidin-3-glucoside and the previously calculated monomeric anthocyanin pigment in milligrams per liter converted to moles in the sample. The ratios were then used to calculate the amount of moles of caffeic acid required to obtain the necessary anthocyanin–caffeic acid molar ratios. This was then converted to grams using caffeic acid’s molar mass and combined with 27 mL of EtOH. These EtOH samples with or without caffeic acid were added to 108 mL of Phosphate Citrate buffer and 42 mL of ACN in Phosphate Citrate buffer.

#### 3.2.4. Incubation

To measure the effect of temperature, samples were placed in Fisher Scientific Isotemp 637D incubators (Thermo Fisher Scientific, Waltham, MA, USA) at temperatures of 25 °C, 50 °C, and 75 °C. At hours 0, 3, 6, 24, 48, and 72, samples were removed from incubators, filtered using 0.3 micromolar filters, and frozen in uHPLC vials until analyzed. Samples were removed from the freezer and left at room temperature for 15 min until thawed.

To measure effect of caffeic acid, samples were placed in Fisher Scientific Isotemp 637D incubators (Thermo Fisher Scientific, Waltham, MA, USA) at 65 °C. At hours 0, 2, 4, 8, 24, 48, 72, 120, or 168 samples were filtered, frozen, and thawed following the same procedure as above.

#### 3.2.5. Analysis of the Sample

The uHPLC was coupled with a PDA detector and used a Restek reverse phase C-18 column with 50 mm, 2.1 mm, and 1.9 μm size, diameter and particle size respectively (restek Corporation, Bellefonte, PA, USA). The settings were as follows: 0.25 mL/min flow, 50 °C oven temperature, 50 μL injection volume, and the following acetonitrile gradient: 0% at 0 min, 26% at 19 min, 30% at 20 to 21 min, 0% at 21 min.

The SpectraMax M2 spectrophotometer (Molecular Devices, Sunnyvale, CA, USA) was used with a standard 96-well microplate to obtain UV-Vis spectra and 35 μL of each sample was pipetted into separate wells and measured from 380 nm to 720 nm in 1 nm intervals with a 10-degree observer angle, regular transmission, and a D_65_ illuminant. The software ColorBySpectra was used to convert the UV-vis spectral data into color spaces data.

#### 3.2.6. Statistical Analysis

Data analysis to determine the statistical significance of research findings was performed. Microsoft Office 2019 Excel (Redmond, WA, USA) was used to calculate averages and standard deviations. It was also used to develop figures and tables. All statistical data were analyzed using RStudio (Boston, MA, USA). Linear relationships, parallel slope assumptions, and homogeneity of variance were tested between covariates and temperature and absorbance. Both one-way ANOVA and uncentered ANCOVA models were run between covariate, absorbance, and temperature (2-tailed, α = 0.01).

#### 3.2.7. Treatment Performance Equations

These equations quantified the performance efficiency of the pigments by determining the percent of PACN that formed, the percent of pigment that was PACN, and the amount of pigment which remained.

PACN yield percent = [(AUC_500–520 nm_ C3S PACN + C3G PACN t_n_) ÷ (AUC_500–520 nm_ C3S + C3G at Day 0)] × 100 (3)

Percent PACN to total pigment percent = [(AUC_500–520 nm_ C3S PACN + C3G PACN t_n_) ÷ (AUC_500–520 nm_ C3S +C3G + C3S PACN + C3G PACN at t_n_)] × 100 (4)

Remaining pigment percent = [(AUC_500–520 nm_ C3S + C3G + C3S PACN + C3G PACN t_n_) ÷ (AUC_500–520 nm_ C3S + C3G + C3S PACN + C3G PACN at t_0_)] × 100 (5)

AUC = Area under the Curve, C3S = cyanidin-3-sambubioside, C3G = cyanidin-3-glucoside, PACN = Pyranoanthocyanins from C3G and C3R.

## 4. Conclusions

The use of heat was effective for increasing PACN content. We found the use of heat and caffeic acid showed PACN increased at a faster rate over time than without these applications. We found significant evidence of this between 25 °C and 75 °C. Incubation of samples at 25 °C for 24 h retained the most pigment (98%) while incubation of samples at 75 °C for 72 h retained the least pigment (30%). However, at 75 °C incubation of samples, 90% converted to PACN within 24 h and 100% within 48 h. Because of pigment degradation over time, incubating for 24 h at 75 °C may be more beneficial under these conditions to retain the pigment. At high incubation temperatures, the increase in caffeic acid concentration increased PACN content consistently, but not significantly. This brings into question the validity of the increasing caffeic acid due to the negative organoleptic effects it may pose to the finished product. The difference between no caffeic acid and the addition of caffeic acid was not significant in the amount of pigment remaining, although the samples containing caffeic acid had a slightly greater pigment remaining. Of the pigment remaining, the sample with caffeic acid would have a longer lasting and more stable pigment because of the PACN content. There was a correlation between the increase of PACN and the decrease of anthocyanins. The addition of heat, however, drastically decreased the amount of pigment that remained over time. Future work should consider how these results may be applied to the food industry.

## 5. Patents

Patent pending. P2020-226-5636.
